# 

**Published:** 1854-07

**Authors:** 


### INDEX TO VOLUME II.

| | Page. |
|---|---|
| Abdomino-Renal, | 153 |
| Acetate of Iron, | 167 |
| American Med. Association, | 464 |
| Anasarca, | 325 |
| Anatomy and Physiology, | 57 |
| Anæsthesia, Local, | 481 |
| Albugo, | 485 |
| Auscultation, Introduction, | 462 |
| Auscultation, The Practice of, | 286 |
| Belladonna, | 315—407 |
| Blood-letting, | 39—76 |
| [illegible]muth, Subnitrate, | 488 |
| [illegible]gies, Sponge, | 484 |
| Calomel in Fever, | 328 |
| Calculus, Bilateral operation, | 476 |
| Carbuncles and Boils, | 408 |
| Cases and Observations, | 434 |
| Chloroform, | 160—487 |
| Chlorosis, | 488 |
| Clavicle, Luxation, | 245 |
| Correspondence, | 9—89—169—273 |
| " " | 331—426—441 |
| Cod-liver-oil, | 54 |
| Constipation, | 379 |
| Congenital Obstruction, | 401 |
| Correction, | 151 |
| Croup, Membranous, | 479 |
| Death from Laudanum, | 406 |
| Delirium Tremens, | 248 |
| Death of American Physicians, | 375 |
| Disease of the Eye, | 37 |
| Dr. A. P. Eliston, | 376 |
| Dr. Beale's case, | 468 |
| Dr. Drake and his Critics, | 49 |
| Dr. Drake's Work, | 470 |
| Dr. Samuel Brown, | 175 |
| Dr. E. Williams, | 148 |
| Eclampsia, Case of, | 102 |
| Elkoplasty, | 297 |
| Ergot | 324 |
| Evansville Med. School, | 376 |
| Epilepsy, | 488 |
| External Heat and Cold, | 483 |
| Fever treated Homœopathically, | 467 |
| Foreign Bodies in Air-passages, | 470 |
| Gleanings Abroad, | 78 |
| Gangrene, Dry, | 183 |
| Gangrene of the Lung, | 328 |
| Gout, | 480 |
| Gum Mezquite, | 437 |
| Headache, Neuralgic, | 163 |
| Hints for Young Doctors, | 322 |
| Healthy Skin, | 353 |
| Hemoralopia, | 387 |
| Heat, Observations on Exhaustion, | 68 |
| Hemorrhoids, | 105 |
| Health of Emigrant ships, | 147 |
| Influence of the Mind on Disease, | 407 |
| Introductory Address, | 409 |
| Iodine in Leucorrhœa, | 388 |
| Iodine, Topical uses, | 404 |
| Institutions for the Insane, | 223 |
| Ipecacuanha, | 246 |
| Laryngitis, | 486 |
| Leucorrhœa, | 485 |
| Laboratory, Prof. Smith's | 374 |
| Lupus, | 485 |
| Lectures of the Blood, | 466 |
| Law of Cholera, | 391 |
| Lectures on Fevers, | 303 |
| La Roche on Pneumonia, | 125 |
| Malformation, Genital organs, | 23 |
| Milk-sickness, | 25 |
| Medical Profession and Politics, | 45 |
| Medical Appointments, | 47 |
| M. Roux, | 55 |
| M. Velpeau, | 158 |
| Medical Society of Kentucky, | 296—363 |
| Mastodon Giganteus, | 288 |
| Miscellaneous cases, | 289 |
| Medical Testimony, | 226 |
| Meteorological Table, | 152—232 |
| Medical Association, N. Y. | 346 |
| New Publications, | 470 |
| Nitric Acid, | 377 |
| Olea Europœa, | 408 |
| Oil of Turpentine, | 482 |
| Ophthalmoscope, | 533 |
| Poisoning, | 480—486 |
| Precocious Infant, | 407 |
| Prof. Flint's Introductory, | 469 |
| Prof. Gibson, | 375 |
| Prof. S. B. Hunt, | 375 |
| Pestilence and War, | 318 |

| | Page. |
|---|---|
| Protracted Pregnancy, - - | 318 |
| Per-Chloride of Iron, - - | 316 |
| Pulmonary Tuberculosis, - | 211 |
| Pyrosis, - - - - - | 161 |
| Prof. Tiedemann, - - - | 55 |
| Prof. T. R. Beck, - - - | 56 |
| Placenta Previa, - - - | 86 |
| Prolapsus Uteri, - - - | 100 |
| Phlebitis, - - - - - | 484 |
| Quinidine in Fever, - - | 319 |
| Quartain, Obstinate, - - | 77 |
| Rheumatism, Chronic, - - | 486 |
| Rupture of the Uterus, - - | 402 |
| Rape on an Idiot, - - - | 383 |
| Richardson's Anatomy, - - | 149 |
| Strychnine, - - - - | 483 |
| Surgery, - - - | 27—477 |
| Skeleton and the Teeth, - - | 469 |
| Salivary Fistula, - - - | 439 |
| Sunstroke, - - - - - | 85 |
| Soda Water, - - - - - | 84 |
| Spirit Rappings, - - | 292—314 |
| Suture of Tendons, - - | 248 |
| Salt, Non-necessity of, - - | 168 |
| Syphilitic Diseases, - - | 218 |
| Tumor, Fibrous, - - - | 487 |
| Tinea, - - - - - - | 487 |
| The Season and its Diseases, - | 18 |
| Tetanus, Traumatic, - - | 329 |
| Types of Mankind, - - | 186 |
| The Weather, - | 151—230—374 |
| Unpublished Reports, - - | 254 |
| University of Louisville, - | 473 |
| Viviparous Fish, - - - | 427 |
| Variola and Vaccina, - - | 327 |

